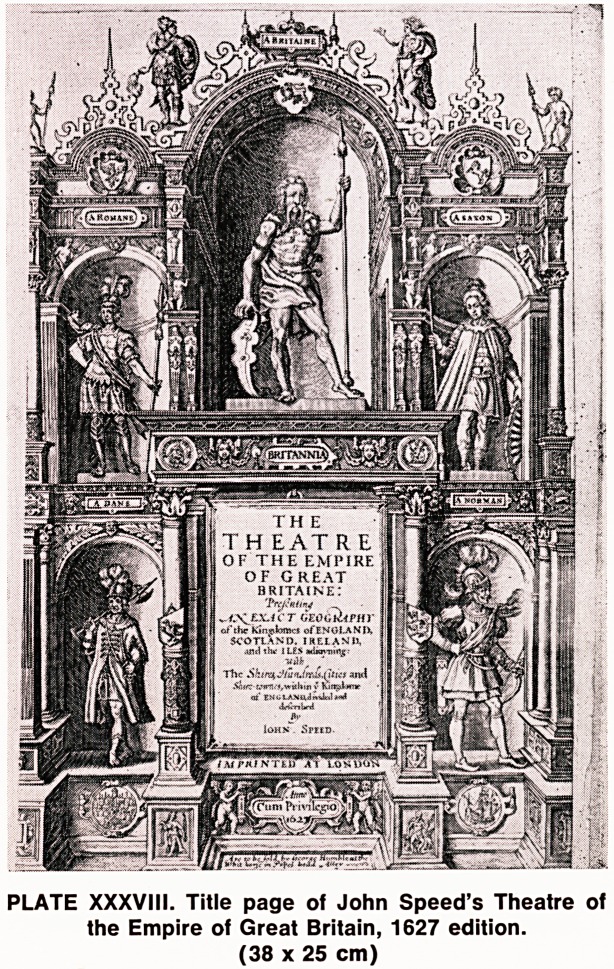# County Maps of the West Country

**Published:** 1971-07

**Authors:** J. P. Mitchell


### County Maps Of The West Country

J. P. Mitchell, M.S., F.R.C.S.

The squirrel instinct is inherent in most of us from the earliest age, and the reason for this desire to hoard is often obscure. It is not necessarily the miser in us, storing property in the hopes of increasing our worldly assets, as many will collect items which have little or no intrinsic value. The schoolboy's interest in match box covers, cigarette cards and a host of other curiosities, which have been illustrated from time to time in Cliff Morgan's television programme of children's hobbies, are perfect examples of collections of worthless items.

Maps are probably one of the oldest attractions to the collector, but the earliest collections were only for the nobility of considerable wealth, such as Kings, Emperors and highly successful military commanders. Today, modern accurate maps are extremely cheap, considering the intrinsic detail in the cartographic preparation.

My interest in collecting maps evolved through philately. A gift of a small collection of foreign postage stamps combined with an opportunity to search for envelopes at my father's office which, at that time, had an enormous world-wide correspondence, gave me a flying start for a schoolboy's stamp collection. The diversity of envelopes and postmarks was intriguing and some of the countries of origin could only be identified in the larger and better atlases. This collection developed on the principle of the number of countries represented, rather than just the acquisition of as many stamps as possible. It became an obsession to have

**PLATE XXIV. Wiltshire by Christopher Saxton 1576 (47.5 x 41.5 cm)**

every known country represented by a few stamps and soon the addition of a small map, identifying the more obscure countries was the next interest. The best map that could be found was inserted on the page in front of the stamps of each country.

Old atlases could be bought at secondhand bookshops for very little money and the collection of representative maps became as fascinating as the stamps themselves. This interest in cartography progressed to the quest for older maps, which were then relatively inexpensive and, since the war, my small collection of antique maps has crystallised into a more selective interest in the six counties of the West Country. After 1700 the variety of maps became numerous and less attractive and, therefore, I confined the period of my collection to the 16th and 17th centuries.

The earliest known maps of the counties of England and Wales, produced with a measure of accuracy from a careful survey, were those of Christopher Saxton, who was born in Yorkshire in 1542. Having completed his education at Cambridge, he was appointed to the house staff of Thomas Seckford, who was then Master of the Court of Requests, and Surveyor of the Court of Wards and Liveries to Queen Elizabeth. Whether Seckford knew of Saxton's ability as a cartographer before or discovered it after he was appointed to his household is not known. Nevertheless, he very quickly appreciated Saxton's talents in this direction and gave him authority to survey the counties of England and Wales and paid all expenses. This authority was endorsed by Queen Elizabeth herself, who gave Saxton access to all parts of the countryside and, in particular, to any tall buildings, from which he could make his survey. It is said that he completed this survey in just over two years. These maps are simple and probably some of the most beautiful of the county maps (Plate XXIV). They show the coastline, rivers, hills and a number of towns, but there are no roads and the division of counties into hundreds is not illustrated. These maps have very attractive cartouches, ships and sea monsters. There

**PLATE XXV. Somerset by William Kip 1607 (38 x 28 cm)**

is usually the characteristic scale in miles, surmounted by a pair of dividers. The only coat of arms on the maps of Saxton are those of his patron and benefactor, Thomas Seckford. These maps were issued uncoloured, though delicate and attractive colouring was added in many cases by the original purchaser.

The maps of Saxton were re-issued in a modified form and in a smaller size, approximately half the dimensions of the original map, by William Hole and William Kip working together (Plate XXV). The first issue they produced was in 1607 and had a latin text on the back, the second issue in 1610 had a plain back, and the third issue in 1637 also had a plain back, but in this last issue there is a numeral denoting the number of the map in the atlas. This number appears in the bottom left hand corner of most of the county maps. These maps are almost as attractive as the original Saxtons and, because of their smaller size and larger numbers printed, are consequently much more frequently seen than the original maps by Saxton himself.

In 1611, John Speed, whose name is probably the first to come to mind when most people talk of old maps, produced his finest issue of The Theatre of the Empire of Great Britain, which included county maps of all the counties of England and Wales. His maps were based to some extent on those of Saxton, but he added small pictures of certain features within the county, such as a town plan of the City of Gloucester, and also the City of Bristol in the County map of Gloucestershire (Plate XXVI). The second city of the land was at that time erroneously included, as Bristol had already been granted the status of a county. Speed's maps were the first to include written information on the map, other than the names of towns, rivers and counties. He also included Coats of Arms of various members of the nobility resident in that county. Speed's maps of Oxfordshire and Cambridgeshire are particularly popular as they show the colourful coats of arms of all the colleges. His maps were reprinted in many issues, the details and distinguishing features of these issues being beyond the scope of this article. His maps were issued in book form, as an atlas, with very elaborate illustrations on the first eight pages of the atlas.

A year after John Speed's original issue, another series of maps appeared in the quaintest form: Michael Drayton's Polyolbion (1612). These are attractive little maps with numerous figures depicting the various physical features. There are shepherds either sitting or standing on the tops of the hills, rivers with water nymphs rising from the water like "Ondine", towns depicted by crowned ladies, and Diana and her hounds represent the important hunts of the district (Plate XXVII). These maps contain very little useful information from the point of view of a cartographer, and are not orientated to any particular meridian, but as

**PLATE XXVII. Gloucestershire by Michael Drayton 1612. (34 x 25.5 cm)**

works of art they are delightful and have a worthy place in any collection of maps.

The next interesting series are the many miniature maps, the first of which were produced by Pieter van den Keere, a Dutch engraver who spent most of his time in England. His maps were approximately postcard size and, inevitably, the content and detail were reduced, and the cartouches were much less elaborate (Plate XXVIII). Whether these were intended as travelling maps, being small enough to carry the entire atlas in a large pocket, is still obscure. These maps were later published by John Speed and, as a result, have often been called miniature Speeds, though in fact

**PLATE XXVI. Gloucestershire by John Speed 1610. (50 x 37 cm)**

**PLATE XXVIII. Somerset by Peter Keer. (Pieter van den Keere, 1599) (12.5 x 8.5 cm)**

**PLATE XXIX. Somerset by John Bill 1626. (12.5 x 8.5 cm)**

he himself was not responsible for the design of any of these maps. Later, in 1626, an even more detailed and elaborate series of miniatures were published by John Bill (Plate XXIX). These are much less frequent than the Keere's and, in my opinion, considerably more attractive. The cartouches are no larger but are more detailed, and degrees of latitude and longditude are marked along the margins of these maps.

We now move into the era of the two most famous early Dutch engravers and cartographers, Blaeu and Jansson. Theirs were the first maps to be issued fully coloured by the publisher himself. Other earlier maps were, in the main, issued uncoloured and the colouring was carried out by colourists employed by the purchaser of the atlas. Many of these early maps can still be found in their uncoloured state.

**PLATE. XXX. The Bristol Channell by Blaeu 1623. (35 x 26 cm)**

Blaeu's earliest maps were his sea charts round the English coast (1623) of which the Bristol Channel is shown (Plate XXX). His county maps appeared in 1645 and were published in large numbers. Blaeu's maps also included a full series of the provinces of Scotland. These maps are very similiar to the maps of Speed, except that no town illustrations or paragraphs of information appear on the maps. The heraldry, however, corresponds to that of Speed. They are excellent quality maps and are of a characteristic and easily recognisable design (Plate XXXI). Jansson's maps appeared a year later and are said to be copied from Blaeu. They are not so easily found as Blaeu, being less plentiful on the market. Their design is perhaps a little more detailed and consequently more attractive to the collector (Plate XXXII). It may at times be difficult to distinguish a Blaeu map from a Jansson, unless the purchaser is already aware of the particular design of that county.

(50 x 41 cm)

**PLATE XXXI. Gloucestershire by William Blaeu 1645.**

(51 x 38 cm)

**PLATE XXXII. Gloucestershire by John Jansson 1645.**

In 1675, John Ogilby published his series of road maps. These are almost as well known as the county maps of Speed, and are laid out on the principle of a continuous roll of parchment, much in the same style as the modern A.A. route map (Plate XXXIII). They are very popular and much sought after. The earliest edition is difficult to distinguish, except by the numerals appearing in the corner of the map. These are altered in the later editions. Miniatures of Ogilby's maps were

**PLATE XXXIII. Bristol to Exeter by John Ogilby 1675. (45 x 35 cm)**

also published, but these did not appear until early in the next century.

Although there were many other cartographers of note in the 17th century, it is possible to mention only two more here, Richard Bloom and Robert Morden, both of whose series were published towards the end of the 17th century. They are much simpler maps and smaller in size than Saxton's, Speed's, Blaeu's and Jansson's. Bloom's are of interest and are accurate in detail, but Morden's are more sought after as artistic cartography (Plate XXXIV). Morden's maps were republished in many editions, and the early editions can only be distinguished by the type of paper on which they were printed.

**PLATE XXXIV. Somerset by Robert Morden 1695. (42 x 36 cm)**

**PLATE XXXV. Town Plan of Bristol by Robert Ricart 1479. (15 x 12 cm)**

Collecting maps of the counties of the West Country must include the maps of the City and County of Bristol, of which our local museum has an excellent collection. The earliest map is that of Robert Ricart, dated 1479, which illustrated the four gates of the old City of Bristol, shown in somewhat Italian style (Plate XXXV).

The next historical plan of Bristol is the map published by Braun and Hogenberg in 1581. This town plan under the title of "Brightstowe" (Plate XXXVI) is known to most Bristolians. This appeared in numerous later editions and was subsequently coloured in an attractive style. It is unsigned and has been attributed to William Smith (1568).

**PLATE XXXVI Bristol by Braun and Hogenberg 1564. (44 x 34 cm)**

The first Bristol map to be drawn to scale was that of John Millerd in 1671 (Plate XXXVII). The scale of his original map is probably seven inches to the mile, but there is a larger scale of 14 inches to the mile dated the following year and, of this, two manuscripts exist; one is in the British Museum and the other in the Bristol City Museum. The first of these was discovered by John Pritchard, who gives a very full account of this map (1926). There is a third publication of Millerd's map in 1673, and each of these publications differs, not only in scale, but various other details of description and design. It is in these Millerd maps that the following quotation appears "The City of Bristol stands upon the borders of Somerset and Gloucester, yet belongs to neither, but is a City and County of itself . . . Bristol may now be reckoned the second City of this kingdom, as well as for trade, number of inhabitants and also for it largeness; the greatest port thereof stands in the bounds of Gloucestershire, the rest in Somerset, but in truth it is a shire within itself, the major part is situated between two rivers, Avon and Frome, which, five miles from thence, fall into the Severn." This also accounts for the development of the name "Bristol" from "Brightstop" through "Brightstowe" to "Bristowe" and ultimately

**PLATE XXXVII. Bristol by John Millerd 1671. (24 x 23 cm)**

"Bristol". Even Bristol itself was originally spelt with a double "L" (Bristoll).

Most collectors can recount anecdotes of their mistakes and their successful finds. It is usually dealers who tell how they learnt the hard way, by paying money for either useless trash or a fake, and collectors like to recount how they were successful in finding a valuable treasure in a junk shop. The chances of a lucky find today in map collecting are remote, as most dealers know that old maps are of value and will nearly always check them before offering them for sale. However many mistakes are made in assessing the year of certain editions and here the collector of old maps has to be on his guard that he is not paying the value of a 1610 Speed, when he is receiving only a 1645 edition. With Speed's maps it is relatively easy to identify the dates of publication, but sometimes it is only by the paper on which the map is printed and the quality of the plate, that is to say, the degree of wear on the plate may be the only means by which the collector can prove the year in which a map was actually printed. An example of this is in the series of sea-charts provided by Captain Greville Collins, the original edition of which was in 1695. Some two or three years ago I was trying to identify a Greville Collins map of the river Avon and took it to the British Museum map room to compare with the six known editions, each of which could be seen in this map library. On inspecting the 1695 bound volume at the British Museum, I discovered that the map of the River Avon was missing. The Curator told me that this map could not have been in the original edition as theirs was complete but, after comparing the successive plates of the other Collins sea-charts, we noted that, by the wear on the plate of the later editions of the river Avon map, there must have been a plate in existence in 1695, and somebody in the past had evidently removed this sheet from the British Museum volume. I was very relieved that the Curator himself had been with me when I arrived and while we were examining these maps, otherwise I might personally have been under suspicion.

Sometimes one feels the need to refer to one's own reference notes on maps before being able to identify a map accurately. On one occasion I paid too great an interest in an early anonymous map of Wiltshire, at an Antique Dealer's Fair. The remarks I had made to the dealer and, in particular, to my wife had evidently been overheard by another dealer nearby. I put the map down for a few minutes to sit quietly in a corner and refer to my notes and when I returned, three or four minutes later, it had already been purchased by this other dealer and I had lost what turned out to be a real bargain.

There are already a number of copies of these old maps. Most of them are copies drawn by the British Museum as perfect replicas, but these are clearly stamped to that effect at the bottom of the map. They are very elegant and can be purchased for a modest sum, but as recently as this year I was nearly taken in by an early copy which was being offered for sale at an auction. The map was badly stained and mottled with age, there was no indication that it was a copy, until the plate marks were examined very closely. The map was mounted in an excellent mount and frame, and it was impossible to see the back. Fortunately, I never buy a map of any value without first taking it out of its frame so that the type of paper, the reverse of the map and the watermark can all be examined.

**PLATE XXXVIII. Title page of John Speed's Theatre of the Empire of Great Britain, 1627 edition. (38 x 25 cm)**

It is still an unhappy fact that many beautiful atlases are being broken up to sell the maps individually. Four years ago I came across an atlas which had been sold at Sotheby's, and the dealer who bought it had only recently taken an interest in antique maps. He had already committed the worst possible sacrilege by tearing out some of the maps and selling them individually for a fairly substantial price, but he still had one map in the atlas that I needed for my collection. Having bargained with him for the price of the map, I asked him what he was going to do with the remaining illustrations and all the literature. He explained to me in his ignorance that that part of the atlas was worthless and he would probably throw it away, so for a small sum he agreed to sell the entire text when he had eventually disposed of all the maps. This would include the introductory front of the atlas, one page of which is illustrated in Plate XXXVIII. With some apprehension I waited for the next three weeks, fearing that he might realise the value of these cartouches and other drawings at the beginning of the atlas. When eventually I collected my trophy, some three weeks later, I was more than delighted to find, on arriving back in Bristol, that the dealer himself had known so little about the atlas and its map distribution, that he had left, in the front of the atlas, one other map which, though not saleable as a county map, had an intrinsic value for a collector.

To examine in detail old county maps, there are a number of places worthy of a visit. In the first instance, the local Archives in most large towns and cities have some old maps of the surrounding district. The City Library will contain well illustrated books about the county maps, such as Pritchard's Literature on Bristol. The map room at the British Museum is open all day and a vast collection of antique and contemporary maps are available, though table space for comparing, simultaneously, several editions of a particular map is a little limited, but the patient help available from the Curator and his staff is invaluable. The Libraries of the Royal Geographical Society and the Institute of Chartered Surveyors contain a large number of old maps which can be viewed by special arrangement. Finally, throughout the country there are numerous collectors who, like anyone obsessed with any form of collecting, are only too delighted to show their specimens to an interested observer. There is also a Society of Collectors, known as the Map Circle, which is formed for the exchange of information on cartography.

Many books have been written on the subject of map collecting, both in Victorian times and in the present century, and it would be impossible here to give a comprehensive review, even of the maps of Gloucestershire, Somerset, Wiltshire and the County of Bristol, but a brief bibliography is appended here for anyone whose interest I may have stimulated on this subject which has, to me, been very absorbing for more than forty years.

I must express my thanks to Mr. John Etoff for his detailed attention to the reproduction of the plates and for sacrificing so much of three weekends in preparing them.

**BIBLIOGRAPHY**

(Received for publication Dec. 1970)

CHUBB, T. (1927 & 1969) The printed Maps in the Atlases of Great Britain and Ireland: a bibliography 1579 to 1870.

CHUBB, T. (1911) A descriptive catalogue of the printed maps of Wiltshire. The Wiltshire Archaeological and Natural History Magazine. Vol. 37.

CHUBB, T. (1912) A descriptive list of the printed maps of Gloucestershire. The Transactions of the Bristol Archaeological Society. Supplementary to Vol. 35.

CHUBB, T. (1914) A descriptive list of the printed maps of Somerset. The Somerset Archaeological and Natural History Society, Taunton.

FORDHAM, Sir H. G. (1908) Notes on the cartography of the Counties of England and Wales (Hartford).

LISTER, R. (1965) How to identify old maps and globes (Bell, London).

PRITCHARD, J. E. (1922) A hitherto unknown print of the Great Plan of Bristol by Jacobus Millerd 1673. Transactions of the Bristol and Gloucestershire Archaeological Society. Vol. 44 p.203-220.

PRITCHARD, J. E. (1926) Old plans and views of Bristol. Transactions of the Bristol and Gloucestershire Archaeological Society. Vol. 48, p. 325-353.

RADFORD, P. J. (1965) Antique Maps. (B.A.S. Printers Ltd. Hampshire).

SKELTON, R. A. (1952) Decorative Printed Maps of 15th to 18th Centuries (Spring, London).

TOOLEY, R. V. (1949 and 1961) Maps and Map-makers (Batsford, London).

## Figures and Tables

**PLATE XXIV. f1:**
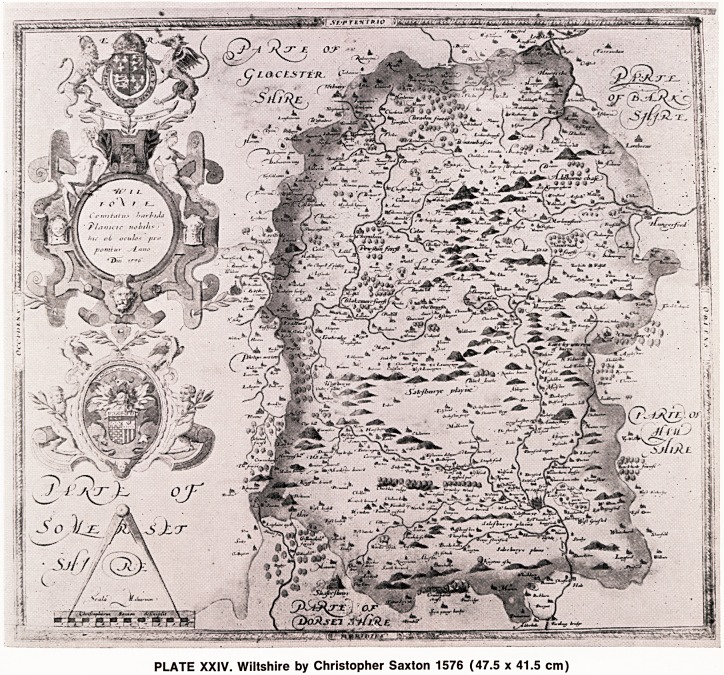


**PLATE XXV. f2:**
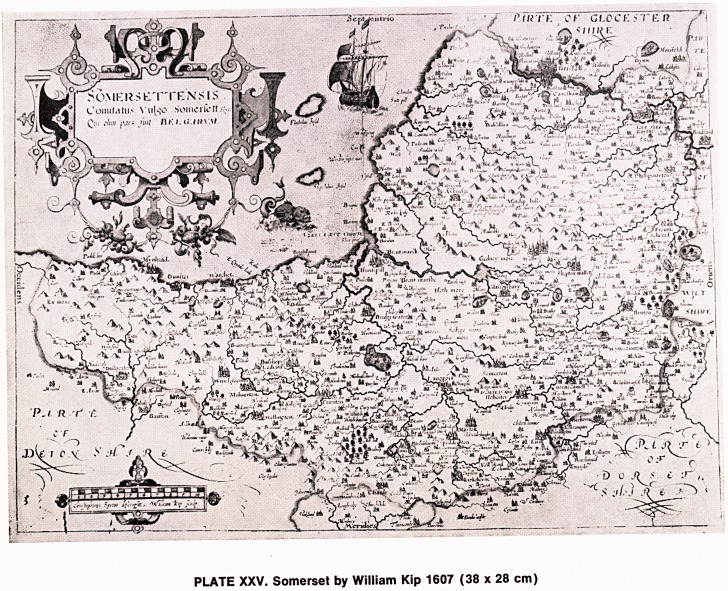


**PLATE XXVI. f3:**
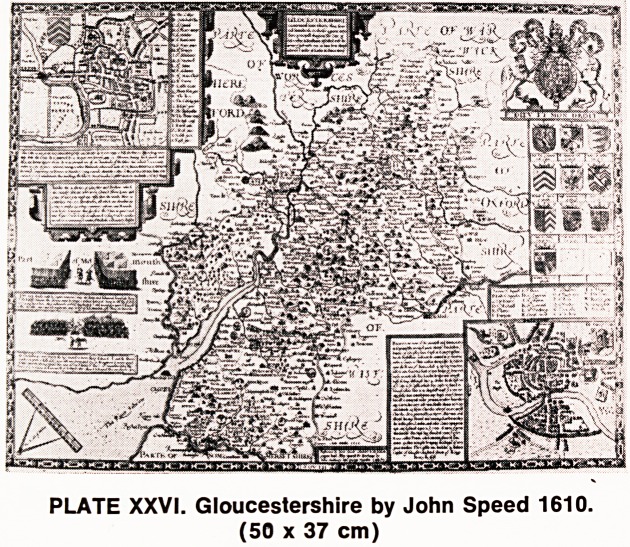


**PLATE XXVII. f4:**
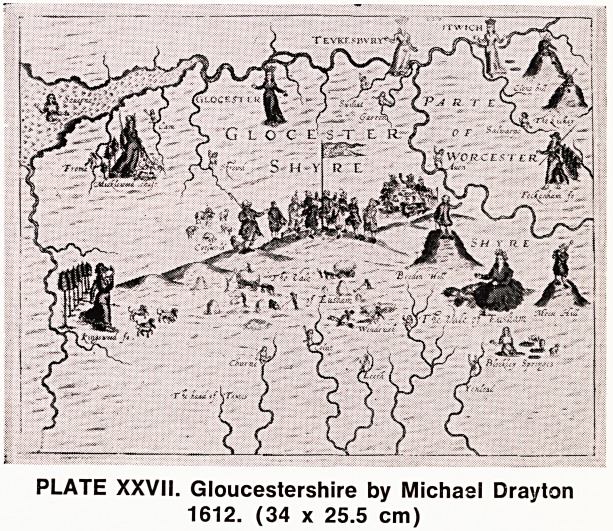


**PLATE XXVIII. f5:**
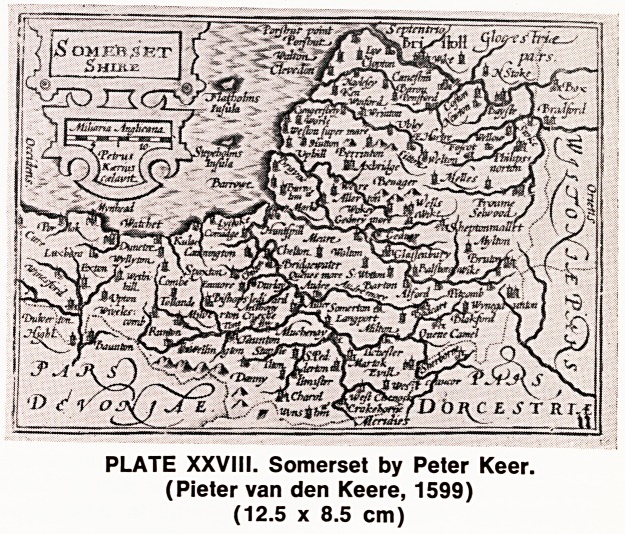


**PLATE XXIX. f6:**
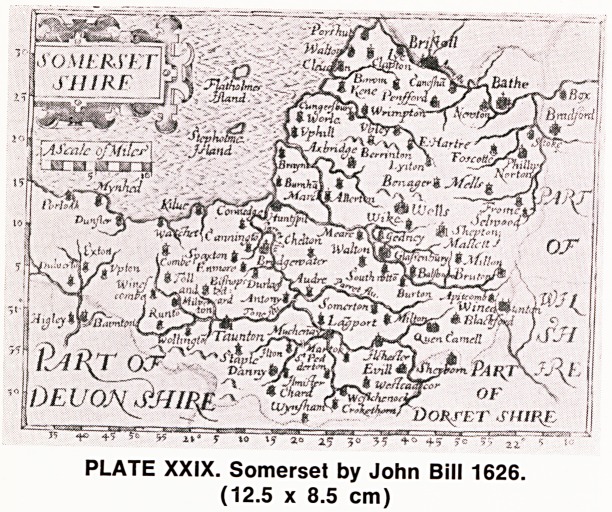


**PLATE. XXX. f7:**
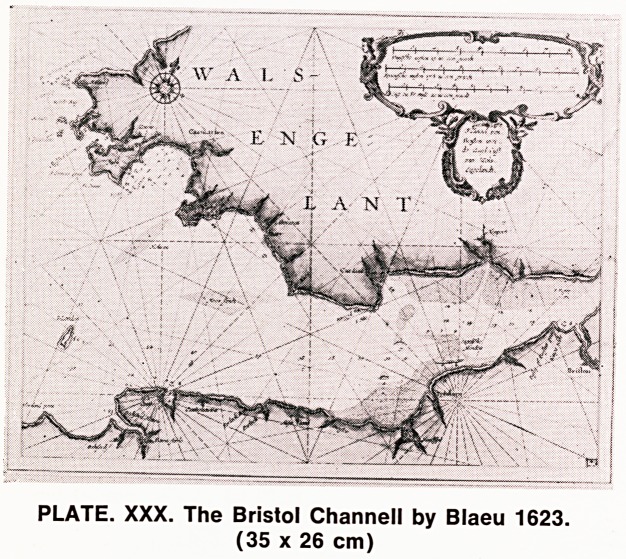


**PLATE XXXI. f8:**
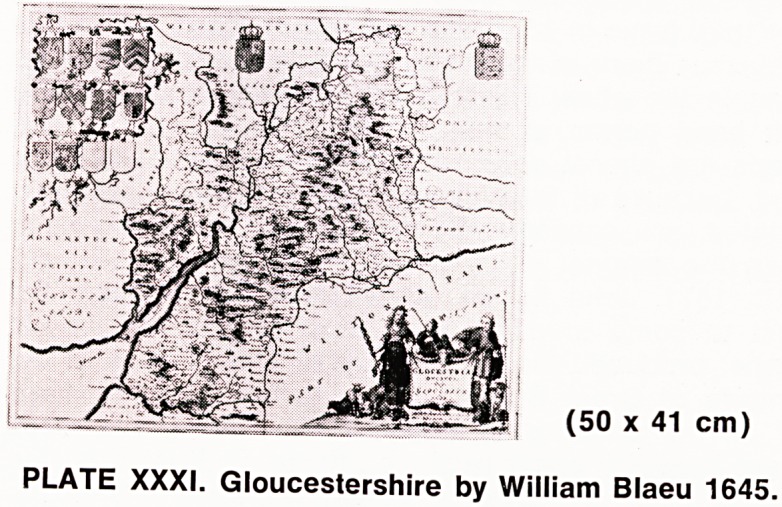


**PLATE XXXII. f9:**
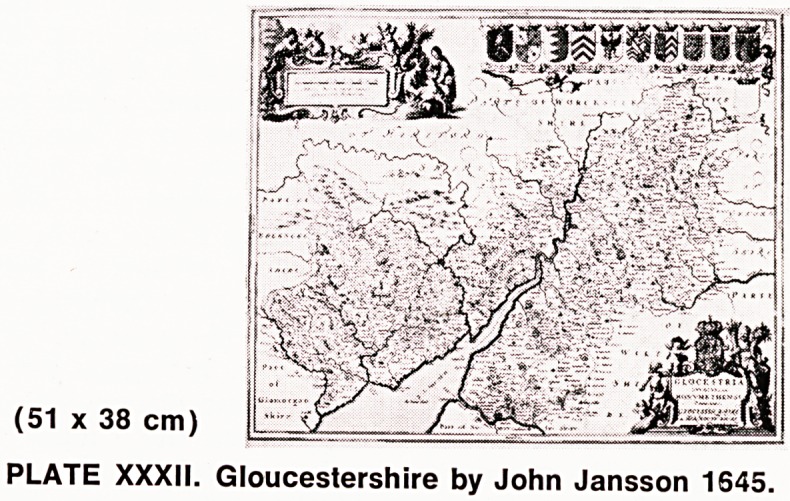


**PLATE XXXIII. f10:**
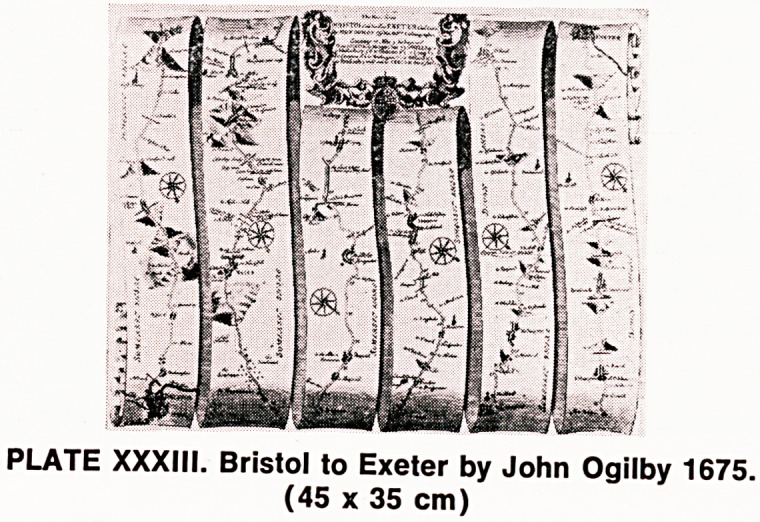


**PLATE XXXIV. f11:**
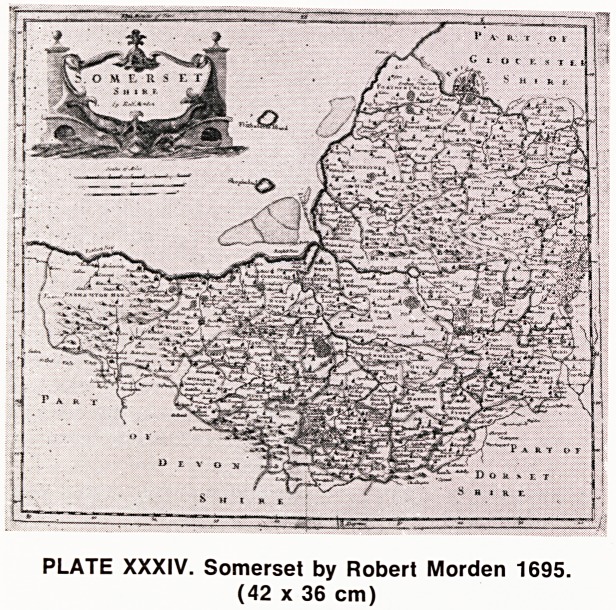


**PLATE XXXV. f12:**
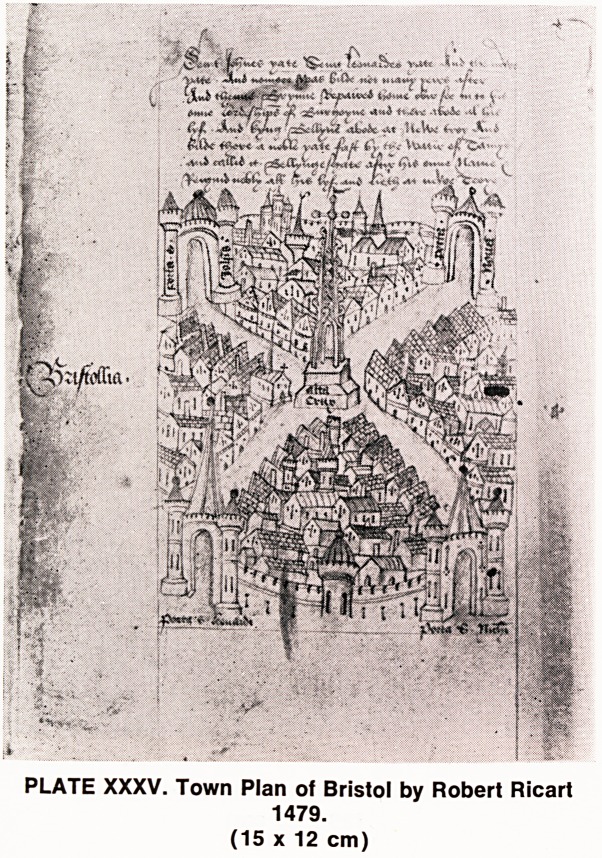


**PLATE XXXVI f13:**
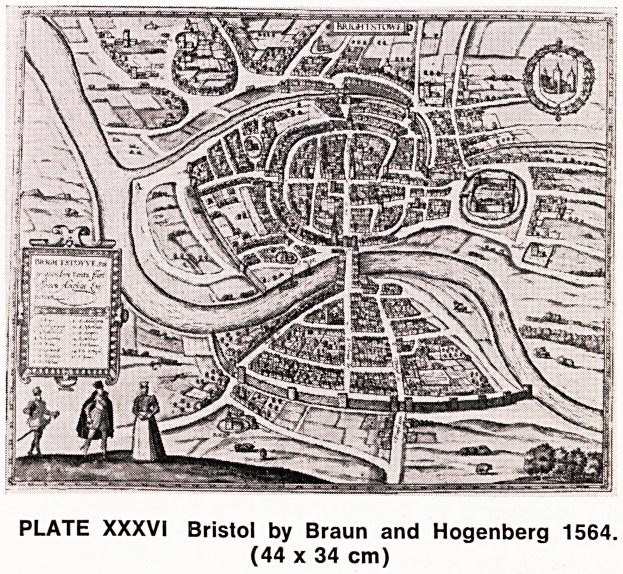


**PLATE XXXVII. f14:**
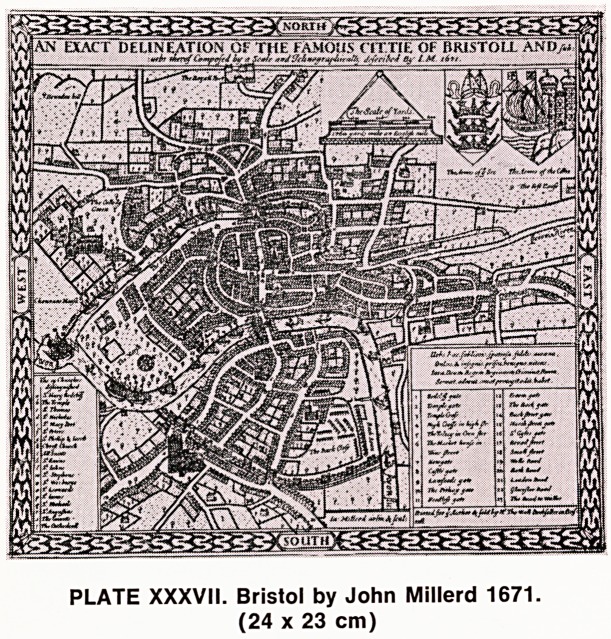


**PLATE XXXVIII. f15:**